# “A Two-Edged Sword”: Paternalistic Leadership and Nurses Performance: A Moderated Mediation Model

**DOI:** 10.3389/fpsyg.2021.775786

**Published:** 2021-12-22

**Authors:** Samyia Safdar, Shazia Faiz, Namra Mubarak

**Affiliations:** Department of Management Sciences, Capital University of Science and Technology, Islamabad, Pakistan

**Keywords:** paternalistic leadership, self-efficacy, power distance, nurses performance, China

## Abstract

**Background:** The study investigates the impact of paternalistic leadership on the performance of nurses. Furthermore, it looks into the role of self-efficacy as a mediator in the relationship between paternalistic leadership and performance. It also looks into the role of power distance as a moderator.

**Methods:** The study used a quantitative survey-based research approach, with questionnaire responses collected over time. Initially, 315 Chinese nurses were surveyed about their views on paternalistic leadership, self-efficacy, and power distance. While their supervisors were called after 6 weeks for a dyadic answer, they were asked to offer their thoughts on their performance. The AMOS 22 software was used for confirmatory factor analysis (CFA), while the SPSS 22 software was employed for descriptive statistics, such as the correlation and regression analysis.

**Results:** The findings demonstrated that paternalistic leadership had a beneficial impact on performance. Furthermore, the role of self-efficacy as a mediator and power distance as a moderating mediator in this relationship has been evidenced.

**Conclusion:** The results suggest that paternalistic leadership has a good impact on nurse performance. Furthermore, self-efficacy as a mediator explains the association between paternalistic leadership and nursing performance adequately. Furthermore, power distance appeared to be a powerful moderator, as the moderated mediation results revealed that in high-power-distant societies, such as China, self-efficacy enhances the link between paternalistic leadership and nursing performance. Limitations and future directions were also discussed.

## Introduction

For the past two decades, researchers have been interested in paternalistic leadership (Jackson et al., 2013; Hiller et al., 2019; Zheng et al., 2020; Khorakian et al., 2021). In the healthcare sector, effective paternalistic leadership plays a significant role in achieving desired goals where patients demand and service delivery expectations from nurses are quite high (Bowles and Bowles, 2000; Jackson et al., 2013; James and Bennett, 2021). Paternalistic leadership has been explored as a leader-centered approach in the literature, emphasizing how the conduct and manners of a leader impact the behaviors of his or her followers (Bono and Judge, 2003). As various psychological and environmental elements affect healthcare workers (e.g., nurses), there is a need to investigate this phenomenon with multiple results and outcomes (Chen et al., 2014; Wang et al., 2018). According to Zhang et al. (2021), the performance of nurses has a significant impact on patient satisfaction and overall health. Chinese nurses are confronted with a variety of issues, including workplace violence (Liu et al., 2021), as well as psychological and healthcare issues (Cai et al., 2020). In high-power-distant societies, nurses require high moral leadership, kindness, and authoritativeness (Ugurluoglu et al., 2018; Sungur et al., 2019; Saygili et al., 2020). The term “paternalistic leadership” was coined by researchers to describe this type of management (Farh and Cheng, 2000; Chen et al., 2019; Lau et al., 2019; Bedi, 2020). Paternalistic leaders serve as mentors to nurses, assisting them in reducing stress and improving their performance (Sungur et al., 2019). This would also improve patient care because nurses would be able to perform more efficiently. Pellegrini and Scandura (2006) found that there is a strong bond between supervisors and subordinates since subordinates show obedience and respond quickly to the protection and care of their superiors. Several studies focusing on the banking sector, hospitality, and academics found that paternalistic leadership has an impact on the in-role performance (IRP) of employees as well as their citizenship activities (Podsakoff et al., 2003). However, the literature is scarce in the health sector, particularly, in the field of nurse performance. As a result, the current research will look into the effect of paternalistic leadership on nurse performance.

The relationship between paternalistic leadership and nurse performance is being investigated using self-efficacy of nurses as an underlying explanatory mechanism. Self-efficacy is the conviction of a person in their ability to manage unpredictably difficult events and problems by planning and carrying out certain actions (Bandura, 1995). The behavior of leaders affects self-efficacy of employees (Zheng et al., 2019). While, Mao et al. (2019) suggested that self-efficacy is linked to an improved subordinate performance. As a result, the researchers believe that self-efficacy of nurses mediates the impact of paternalism on their performance.

**FIGURE 1:**
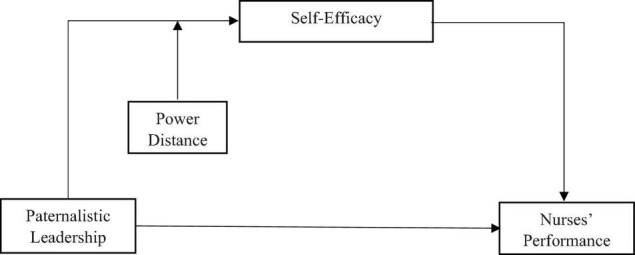
Proposed theoretical framework.

Depending on the cultural context, paternalistic leadership can result in a range of consequences (Chen et al., 2014). China has a high-power-distant culture (Hofstede, 2007), in which people tolerate power distance and are less hostile to the rule of a powerful sect. The current study examines the influence of the style of paternalistic management on the performance of nurses in the presence of self-efficacy as a mediator, using power distance as a moderator (see Figure 1). As a result, the current study adds to the leadership literature in a variety of ways. First, it examines the performance of nurses in the face of paternalistic leadership. Second, it looks into how self-efficacy functions as a mediator between paternalistic leadership and nursing performance. Their attitudes and behaviors are molded by the management style of their bosses, as does their perspective. It also looks into the cultural influence on the claimed phenomenon. Furthermore, as there has been a less research on the performance of nurses in leadership roles, the current study provides useful discussion points.

### Theory and Hypotheses

To better understand the psychological process underlying the influence of paternalistic leadership on the performance of nurses, the current study takes a subordinate-centered approach based on the social exchange and social cognition theory (McCormick, 2001). Individuals look for social resources and try to imitate observable behaviors, such as positive paternalistic leadership, which increases the nursing performance through reciprocal determinism (Banks and Mhunpiew, 2012). According to Gouldner (1960), the social exchange theory is based on the custom of reciprocity or the exchange process, which suggests that people are usually motivated to trade useful acts out of a sense of appreciation and obligation for those who first help them. Thus, the extent to which the behavior of leaders, such as authoritarianism, morality, and benevolence, is enhanced by the exchange process or reciprocation is evaluated in this study. Theory argues that if people have self-efficacy, it will have a positive impact on their surroundings (McCormick, 2001), resulting in better nursing performance. As a result, this study develops a conceptual framework for the relationship between paternalistic leadership, nursing performance, self-efficacy, and power distance.

### Literature Review and Hypotheses Development

#### Paternalistic Leadership and Performance of Nurses

Paternalism makes a positive connection among leaders and subordinates, where the manager/supervisors give the rule to subordinate in his/her own and expert life, and in return, the boss anticipates deference and loyalty (Gelfand et al., 2017; Hiller et al., 2019). However, representative execution is characterized as activities and practices of a worker which are added to the classified objectives and are under his/her control (Rotundo and Sackett, 2002). The extant literature additionally confirmed that leadership impacts the performance of workers (see. Miao et al., 2019; Mubarak et al., 2021). Consistent with hypothetical basics, the social cognitive theory offers a widely inclusive reason for this review (McCormick, 2001). According to the author, the social cognition hypothesis is grounded on the custom of reciprocity or exchange, which prescribes that people are typically headed to craft valuable exercises because of an adroitness of appreciation and obligation toward other people who give initial favor.

In addition, the type of leadership style determines the level of performance of the subordinate (Farh et al., 2014; Ohemeng et al., 2018). In the current study, the researcher examined the comprehensive model of paternalistic leadership and its influence on the performance of nurses using both dimensions of performance, including IRP and extra-role performance (ERP). In general, IRP denotes core job task and responsibilities of employees, which include their job description (Borman and Motowidlo, 1993). ERP of employees is shown by their organizational citizenship behavior (OCB). George and Brief (1992) defined OCB as the behavior of an employee who willingly goes beyond his job and performs activities that are not a formal part of his job requirement.

According to Chen et al. (2014), Confucian moral practice is “higher-ups govern; lower ranks obey.” According to Shu et al. (2018), the behavior of an employee is influenced by the authority of the leader. As part of the Confucian value system, authoritarianism compels employees to obey and comply (Cheng et al., 2014). Few others believe that authoritarian leaders are true motivators for subordinates to go the extra mile (Schaubroeck et al., 2017). However, in Asian culture, these commanding and demanding leadership behaviors contribute to high employee motivation, which has a favorable impact on staff performance. A benevolent leadership style, which exhibits individualized and total concern for the private and domestic comfort of subordinates, is the most valued in Asian culture (Lin et al., 2018). It has a positive impact on the outcomes and performance of subordinates (Li et al., 2018; Huang and Lin, 2021). Morally responsible leaders may encourage employees to engage in social exchange by putting more effort into their jobs and working for their bosses (Kim and Kim, 2013). As a result, this study looked at how the behavior of leaders, such as authoritarianism, morality, and benevolence, can improve the IRP and ERP of nurses.

The social exchange theory strongly supports our assumptions by claiming that the behavior of leaders, such as authoritarianism, morality, and compassion, can improve the IRP and ERP of nurses through an exchange process or reciprocation. Managers who are concerned about control, ethics, and care encourage delegates, and in return, the delegates work with responsibility, demonstrate more visible execution, and show some consideration for others. The ideas are proposed based on the overhead contention.

**H1: Paternalistic leadership has a significant positive effect on the performance of nurses.**

#### The Mediating Effect of Self-Efficacy

The researcher postulated the mediating role of self-efficacy in this connection to quantify the influence of paternalism on subordinate performance. Self-efficacy, as described by Bandura (2010), denotes the confidence of an individual in his/her ability to plan and carry out the steps required to complete a task. The cognitive theory of Bandura focuses on how the behaviors and reactions of an individual are intimately tied to seeing others, and self-efficacy is one of the most important components of it. Knowledge acquisition of individuals is linked to seeing the (model) conduct of other people and the consequences of such a behavior, according to the social cognitive theory (Bandura, 1986). According to the social cognition theory, learning from others is dependent not only on intimate identification between observer and model but also on self-efficacy of an observer. Observers with a high level of self-efficacy, for example, have a better probability of learning through observation. As a result, experiences of followers of witnessing their leader may aid in the development and enhancement of self-efficacy.

According to Shu et al. (2018), authoritative leadership undermines self-efficacy of employees. Self-efficacy of employees is positively influenced by benevolence. Furthermore, Xiaoyu (2010) discovered that moral leadership boosts self-efficacy of employees. As a result, it is plausible to suggest that establishing paternalistic leadership at work could increase self-efficacy of employees. Bracht et al. (2021) provided evidence that self-efficacy is an exact predictor of performance. According to Jacobsen and Bøgh Andersen (2017), leaders can significantly improve employee performance by demonstrating self-efficacy in their subordinates. As a result, it is hypothesized that self-efficacy plays a significant role in explaining the relationship between paternalistic leadership and nursing performance.

Given this review, it is acknowledged that self-efficacy endeavors a key part in the relationship between the paternalistic association and specialist IRP and ERP. The assumptions have strong assistance from the social cognitive theory, as practices are affected by relationships, as such paternalistic pioneers and bosses shape direct of laborers, and finally bring a more critical degree of execution from their side.

*Consequently, it is speculated that*

**H2: Paternalistic leadership has a significant positive effect on self-efficacy.**

**H3: Self-efficacy has a significant positive effect on the performance of nurses.**

**H4: Self-efficacy mediates the relationship between paternalistic leadership and performance of nurses.**

#### Power Distance as a Moderator

The current research looks at the interaction effect of power distance in the leadership-subordinate social exchange relationship. Individuals in high-power distance cultures respect and obey their seniors, and their seniors hold organizational power (Hofstede, 1980). Employees favor paternalistic leaders in cultures with a significant power distance (Bedi, 2020; He et al., 2021). The findings of Hou et al. (2019) are also similar in that the cultures which are high in power distance commonly practiced paternalistic leadership. They claimed that cultures with a greater power distance are accepted more in power imbalances. However, in Western societies, which are low in power distance, paternalism is not accepted and hence criticism results because of inequality in power (Aycan, 2006).

Paternalistic leadership has a favorable impact on employee attitudes in high-power-distant societies (Schaubroeck et al., 2017). In a high-power-distant culture, leaders adopt an authoritative approach that results in improved performance (Wang and Guan, 2018; Chiang et al., 2021). Sue-Chan and Ong (2002) discussed that individuals of high-power-distant cultures find assigned goals effective in increasing their self-efficacy beliefs because they believe that assigned goals are communicated from an authoritative source. As a result, it is proposed that self-efficacy has a strong mediating influence in the relationship between paternalistic leadership and nurse performance at high power distances. The following hypothesis is offered based on the previous arguments.

These assumptions have strong assistance from the social cognitive theory, as it suggests that man is a social animal who recognizes the command, as such if consistent association and persuasive energies come from individuals, then self-efficacy of workers in the presence of kind, caring organization increases and it prompts extended execution. In light of the above contentions, following hypothesis is proposed.

**H5: Power distance moderates the relationships between paternalistic leadership and the performance of nurses through self-efficacy in such a way that the higher is the power distance, the stronger is the mediating effect.**

## Materials and Methods

### Participants and Procedures

It is a time-lag study; with a research philosophy of positivism, the health sector was targeted. A purposive sampling strategy was utilized. A total of 430 questionnaires, utilizing z power, were circulated among medical staff, specifically nurses. Out of which, 315 precise and complete reactions with a response rate of 73% were gathered back for examination. Nurses of public and private hospitals of China specifically Beijing and Tianjin were reached actually for information assortment. A total of 10 hospitals were targeted for the study. For a legitimate reaction, dyadic information was gotten for the presentation of the representative from their supervisors following a gap of about a month and a half. Both the administrators (e.g., leaders, supervisors, bosses) and medical attendants (e.g., nurses) were given a briefing about the nature and motivation behind the examination, and privacy was guaranteed for the authentic information assortment. The dyad poll comprised two sections, i.e., the nursing staff evaluated the paternalistic conduct of their chiefs, their self-efficacy level, and their viewpoint about culture, while supervisors assessed the performance of their nursing staff. The proportion of dyadic reactions was 1:3.

The demographic analysis demonstrates that 38.9% of male and 61.1% of female medical attendants were the members. Of them, 30% of medical staff aged between 20 and 30 years, 45% aged between 31 and 40 years of age, and 24.1% aged 41 years or more. Approximately, members with less than 1-year experience were 12.9%, with 1–3 years were 22.6%, with 4–6 years were 17%, with 7–10 years were 17.7%, and 10 years or more were 29.6%.

### Measurements

#### Paternalistic Leadership

The 26-item scale of paternalistic leadership established by Cheng et al. (2004) was used to measure paternalistic leadership on the 7-Likert scale. Few sample items include “My supervisor is like a family member when he/she gets along with us” and “My supervisor asks me to obey his/her instructions completely.” The Cronbach’s alpha was 0.81.

#### Self-Efficacy

The 10-item scale of self-efficacy developed by Schwarzer (1993) was used to measure self-efficacy of employees on a 7-Likert scale. The sample items of the employee scale of self-efficacy were “I can always manage to solve difficult problems if I try hard enough,” and “If someone opposes me, I can find the means and ways to get what I want.” The Cronbach’s alpha of self-efficacy was 0.92.

#### Power Distance

The 6-item scale of Dorfman and Howell (1988) was used to measure power distance on a 7-Likert scale. The sample items were “Managers should make most decisions without consulting subordinates,” and “It is frequently necessary for a manager to use authority and power when dealing with subordinates.” The Cronbach’s alpha of power distance was 0.86.

#### Performance of Nurses

Two dimensions, namely, IRP and ERP were measured. For IRP, the 6-item scale of Williams and Anderson (1991) and, for ERP of employees, the 20-item scale of Farh et al. (1997) were used on a 7-Likert scale. The sample items of this scale included “This employee fulfills all the responsibilities specified in his/her job description” and “Willing to stand up to protect the reputation of the company.” The Cronbach’s alpha was 0.95.

#### Control Variables

Demographics, such as age, gender, and work experience of leaders, were used as control variables because the current study includes ANOVA that is aligned with old research evidence of their association with employee performance (Ka Sarvari et al., 2016).

## Results

### Descriptive Statistics

The mean, standard deviation, and correlation between variables are shown in Table 1. The study found that all correlations between variables were less than 0.70, indicating that there was no multicollinearity in the data. The results indicate that paternalistic leadership has a significant and positive correlation with self-efficacy (*r* = 0.38, *p* < 0.001), power distance (*r* = 0.37, *p* < 0.001), and performance of nurses (*r* = 0.39, *p* < 0.001). Moreover, self-efficacy is significantly and positively correlated with power distance (*r* = 0.29, *p* < 0.001) and performance of nurses (*r* = 0.52, *p* < 0.001). Meanwhile, power distance is significantly and positively correlated with the performance of nurses (*r* = 0.25, *p* < 0.001). Cronbach’s alpha is used in the SPSS software to determine the reliability of the scale. Cronbach’s alpha is shown on the diagonal, indicating that the data were reliable and suitable for further analysis.

**TABLE 1:** Mean, standard deviation, correlation.

		Mean	SD	1	2	3	4	5	6	7
1	Leaders’ gender	1.41	0.49							
2	Leaders’ age	2.05	0.65	−0.03						
3	Leaders experience	4.03	1.12	0.03	0.56**					
4	Paternalistic leadership	4.64	0.45	−0.05	−0.09	0.01	**(0.81)**			
5	Self-efficacy	5.52	0.68	0.02	−0.08	−0.02	0.38**	**(0.92)**		
6	Power distance	6.11	0.37	−0.07	−0.02	0.01	0.37**	0.29**	**(0.86)**	
7	Nurses’ performance	5.96	0.75	−0.10	−0.10	0.04	0.39**	0.52**	25**	**(0.95)**

*SD = Standard Deviation, Reliability is given on Diagonal.*

**P < 0.01.

### Confirmatory Factor Analysis

Using the AMOS software, confirmatory factor analysis (CFA) was run to further examine the distinctiveness or validity of the variables of scales. The hypothesized baseline model (χ^2^ = 2769.05, *p* ≤ 0.001, χ^2^*/df* = 1.79, comparative fit index = 0.916, root mean square error of approximation = 0.049, Tucker-Lewis Index = 0.912, standardized root mean square residual = 0.049) fits the data well, as shown in Table 2. Table 2 also provides the results of the model comparison, which reveal that other models have poor fit statistics, indicating that the proposed model is suitable for regression analysis and has discriminant validity. In addition, average variance extracted (AVE) and square root value of AVE were used to assess discriminant and convergent validity. The AVE value of all four variables exceeded the threshold value of 0.50, according to the results. Furthermore, the square root of AVE values is bigger than the correlation of latent variables. As a result, the AVE and the square root of AVE results reveal that convergent and discriminant validity exist.

**TABLE 2:** Measurement models comparison of variables.

Models	χ ^2^	*Df*	χ ^2^/*df*	CFI	RMSEA	TLI	SRMR
Null model (Independence model)	15,940.88	1,653	9.64				
Baseline model	2,769.05	1,569	1.79	0.916	0.049	0.912	0.049
Model 1 (when power distance and self-efficacy are combined)	3,889.73	1,579	2.46	0.838	0.068	0.831	0.07

*CFI, comparative fit index; RMSEA, root mean square error of approximation; SRMR, standardized root means square residual; TLI, Tucker–Lewis’s index.*

### Statistical Path Analysis

#### The Main Effect of Paternalistic Leadership on the Performance of Nurses (Hypothesis 1)

Table 3 shows the results of the direct and mediation analyses. Hypotheses 1 and 2 were tested using Model 4 suggested by Preacher and Hayes (2005). The results of direct effect reveal that paternalistic leadership is significantly and positively (β = 0.33, *p* < 0.01) connected to the performance of nurses after controlling for gender, age, and experience of leaders, thus supporting hypothesis 1.

**TABLE 3:** Direct and indirect effect.

Direct effect	Estimate	S.E	*T*
Leaders’ gender	–0.16	0.07	−2.3
Leaders’ age	–0.12	0.06	−1.9
Leaders’ experience	0.07	0.03	2.08
Paternalistic leadership→performance	0.33**	0.08	3.95
Paternalistic leadership→self-efficacy	0.56**	0.07	7.15
Self-efficacy→performance	0.49**	0.05	8.83
**(95% Bias corrected confidence interval method)**			
Indirect effect	Effect	S.E	LL-UL
Paternalistic leadership→self-efficacy→performance	0.27	0.10	0.09–0.49

N, 315; **p < 0.01.

*LL, Lower limit; UL, Upper limit; S. E, Standard error.*

#### The Effect of Paternalistic Leadership on Self-Efficacy of Nurses (Hypothesis 2)

Hypothesis 2 was tested using Model 4 suggested by Preacher and Hayes (2005), and the findings are shown in Table 3. The data show that paternalistic leadership is significantly and positively (β = 0.56, *p* < 0.01) related to self-efficacy of nurses after controlling for gender, age, and experience of leaders, hence supporting hypothesis 2.

#### The Effect of Self-Efficacy on the Performance of Nurses (Hypothesis 3)

Model 4 suggested by Preacher and Hayes (2005) was used to test hypothesis 3, and the findings are shown in Table 3. The data show that self-efficacy is significantly and positively (β = 0.49, *p* < 0.01) related to the performance of nurses after controlling for gender, age, and experience of leaders, hence confirming hypothesis 3.

#### The Mediating Role of Self-Efficacy Among Paternalistic Leadership and Performance of Nurses (Hypothesis 4)

When gender, age, and work experience of leaders were controlled, Model 4 results for mediation as suggested by Preacher and Hayes (2005) revealed that paternalistic leadership had a substantial positive influence on self-efficacy (β = 0.56, *p* < 0.01), as well as a significant and positive effect on performance (β = 0.49, *p* < 0.01). The indirect effect of paternalistic leadership on performance through self-efficacy revealed a significant positive effect (indirect effect = 0.27, 95% lower limit = 0.09, 95% upper limit = 0.49). As a result, the findings show that paternalistic leaders increase self-efficacy of employees and that self-efficacy leads to better performance, thus supporting hypothesis 4.

#### Moderated Mediation Effect of Power Distance Among Paternalistic Leadership and Performance of Nurses (Hypothesis 5)

Hypothesis 5 states that high-power distance strengthens the indirect influence of self-efficacy on the relationship between paternalistic leadership and nursing performance. Model 7 suggested by Preacher and Hayes (2005) was used to investigate the moderated mediation effect of power distance.

**FIGURE 2:**
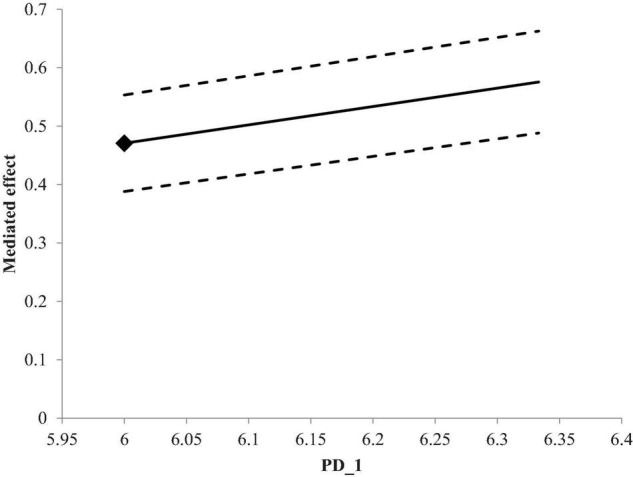
Interactive effects of paternalistic leadership and power distance on self-efficacy.

Results in Table 4 indicate that the effect of paternalistic leadership on the performance of nurses through self-efficacy is positive for high-power distance (effect = 0.28, *p* < 0.01). They also showed that the value of confidence interval for high- and low-power distance effect did not exceed zero, indicating the acceptance of hypothesis 5. Results revealed that the mediating effect of self-efficacy is stronger for high-power-distant societies (see Figure 2).

**TABLE 4:** Regression for testing power distance moderation effect for self-efficacy and nurses’ performance.

IV	DV	a1	a3	b	c′	w	(a1+a3*w) b	CI (L–U)
Paternalistic leadership	NP	0.56**	0.31**	0.49**	0.33**	Low	0.23** 0.28**	0.80, 0.29
						High		

(a1+a3*w) b, Conditional indirect effect of independent variable on dependent variable Via mediator at different levels of moderator.

*a1, Effect of independent variable on Mediator.*

*a3, Effect of interaction between independent variable and moderator on Mediator.*

*b, Effect of Mediator on dependent variable.*

*c′, Direct effect of independent variable on dependent variable.*

*PL, Paternalistic Leadership, NP, Nurses’ Performance.*

*W, Moderator, IV, independent variable, DV, dependent variable.*

*CI (L–U), 95% upper and lower confidence interval with 5,000 bootstrap samples for the index of moderated mediation.*

*p < 0.05, **p < 0.01.

## Discussion

The current findings broaden the work on paternalistic authority by investigating the interceding and directing impacts in the relationship with execution. To the best of the information, this was the main review with exact proof on how the performance of nurses is influenced by the paternalistic authority within the sight of self-efficacy in Asian culture.

### Paternalistic Leadership on Performance of Nurses

The current study uncovered that paternalistic administration has a huge and beneficial outcome on exhibition of medical caretakers. The outcomes were steady with ongoing examinations (i.e., Wang et al., 2018; Hiller et al., 2019; Karakitapoglu-Aygün et al., 2020). Paternalistic administration is a blend of tyranny, ethical quality, and kindness, which praises the standard of incentives. It is a double-sided deal, where pioneers worry about a more significant level of execution, explicitly in the wellbeing area. Accordingly, they offer love and appreciation to their nursing staff, yet keep up with their power well. In this manner, eventually leaving them with no decision for careless conduct, they simply show a sufficient IRP and ERP. An undeniable degree of execution is required because even a slight mix-up can unfavorably influence the patients. Accordingly, supervisors guarantee that with a presented awareness of certain expectations and sensation of worry alongside held control and authority, required execution is acquired.

### Paternalistic Leadership on Self-Efficacy, Self-Efficacy on Performance, and Self-Efficacy as Mediatory Mechanism

Besides, the current examination showed that self-efficacy intercedes the connection between paternalistic initiative and exhibition of medical attendants. The outcomes are an augmentation of the work performed by Bayraktar and Jiménez (2020) where they stressed that foreseeing factors decide the degree of self-efficacy in representatives; furthermore, it helps in accomplishing beneficial results. Our discoveries show that directors with paternalistic styles support self-efficacy of medical staff. Therefore, by showing caring profound quality, authority, and altruism, they upgrade their in-job and extra-job execution. These discoveries are wonderful and add to extant literature because the review is quick to examine the relationship of paternalistic authority of chiefs and presentation of attendants within the sight of intervening variable, i.e., self-efficacy. The thoughtfulness, care, liberality alongside a feeling of moral ethics and guidelines, and a dash of force and authority shows an advantageous degree of order and influential position, eventually reassuring medical attendants to give compelling information, bringing about an expanded degree of execution, simply task-related yield as well as extra-job conduct. Subsequently, it shows a way for the administration that how to oversee staff well. The outcomes are likewise an augmentation of Walters (2021), who inferred the idea that for positive execution levels, self-efficacy is significant.

### The Moderated Mediating Role of Power Distance

The main contribution is the discovery of interactive variable results. Pakistan has been distinguished as a high-power-distant culture where monetary requirements, force, and authority hole have agreeableness (Jehanzeb, 2021). The discoveries indicate that self-efficacy intercedes the impact of the paternalistic initiative on execution when the power distance is high. The current findings thus broaden the extant literature and theory with the input that power-distant social orders have the upgrading impact of self-efficacy on worker execution with regards to authority. These discoveries are interesting as they show the significance of self-efficacy in interpreting pioneer paternalism into presentations of attendants under the umbrella of culture. The discoveries supplement the discoveries of Siddique et al. (2020) that force gets everything it might want; higher positioned and lower positioned people act likewise and can bring inconstancy.

#### Practical and Theoretical Implications for Nursing Management

Nurses in healthcare system of China have faced a slew of concerns over the years, including psychological issues and mental health issues. There is a need to look at the variables that can help to eliminate these consequences and problems. The findings suggest that supervisors, particularly in Chinese hospitals, contribute significantly to the performance of nurses through self-efficacy by showing authority, morality, and benevolence. They exhibit authoritative behavior to ensure timely completion of prescribed tasks, and they are also benevolent. Some academics have previously said that authoritarianism can damage performance, but this study adds to the literature by revealing that overall paternalistic leadership is viewed positively due to its beneficial characteristics, resulting in positive outcomes.

Verifying the research on leadership and self-efficacy of employees, the present study highlights the significance for supervisors to form such a relationship with the employee, which could enhance employee creativity resulting in an enhanced performance. The present study adds several theoretical and practical contributions to the general research stream. First, it suggests that self-efficacy is a critical explanatory mechanism in the relationship between paternalistic leadership and nursing performance, a relationship that has received less attention (Saygili et al., 2020). Employee attitudes as an underlying mechanism in the relationship between paternalistic leadership and employee performance were not adequately studied. The current study reveals that self-efficacy is a key factor in this relationship. It emphasizes the importance of self-efficacy in the leadership process in a country with a high-power distance, such as China. Second, it suggests that paternalistic leaders in high-power-distant cultures should boost self-efficacy of nurses, which would improve their performance. Third, the current study adds a cultural perspective to the literature by looking at self-efficacy in the context of paternalistic leadership and nursing performance.

The research also has some practical implications. To begin with, the findings of this study reinforce the need for paternalistic leadership in the healthcare sector. Nurses work long shifts, which might have a negative impact on their performance. According to this study, hospital administrators can improve nurse performance by ensuring paternalism in their leadership. The paternalistic nature of the leaders should also be considered throughout the hiring process. Also, for those who have already been hired, management can encourage their leaders to act with benevolence and morality in the workplace, which will improve nursing performance. Furthermore, management now understands that authoritarianism in leadership might help nurses perform better. Employees with a combination of positive and negative qualities contribute positively to nurse performance. It is incorrect to focus primarily on its negative aspects. Based on the findings of this study, hospital administrators in high-power-distant societies will be able to improve self-efficacy of nurses, resulting in improved performance, with the support of paternalistic leaders.

### Limitations and Future Research

Even though the current work makes significant contributions, few drawbacks are mentioned. First, paternalistic leadership is a double-edged sword, in that it secures desirable outcomes. As a result, it must be evaluated with different outputs and effects, such as mediators, dependent variables, and moderators. Second, while it is debatable if power distance is perceived as a negative, its positive impact should be investigated across cultures. Third, due to time limits, the study only focused on the health sector; however, future research can focus on a mixed sample for generalization, i.e., by focusing on other sectors and industries. Fourth, the current study argued that authoritarianism has a favorable impact on the performance of high-power-distant employees. Future research should look into the negative effects of authoritarianism on nurse performance in both high- and low-power societies. Finally, eastern and Western cultural impact can all be explored in cross-cultural comparative research; however, we have solely focused on China. As a result, future researchers will have an open window to theoretically and practically evaluate the same model in their setting or a full version of it.

## Conclusion

Paternalistic leadership is a fascinating and innovative topic of research in leadership studies (Lee et al., 2018). Because of its strong and deep roots in Confucian ideology, it has a strong positive impact on Asian societies. The findings of the current study show that the paternalistic leadership of supervisors has a considerable impact on the performance of nurses. They also refute the negative connotations of paternalism and demonstrate its favorable impact on the organizational outcomes and behaviors. Self-efficacy of employees is a major mechanism in elucidating why workers of paternalistic leaders perform well and demonstrate citizenship behavior at work, according to this study, which amassed surplus evidence for the influence of paternalistic leadership. By studying the moderating influence of culture in the association of paternalistic leadership and the performance of nurses, the current study provided new insights into the paternalistic leadership study in the context of culture. The current research created and tested a model that looks at self-efficacy as a mediator and power distance as a moderator in the relationship between paternalistic leadership and nurse performance. The current study provided an interesting analysis of a unified moderated mediation model that increases understanding of paternalistic leadership on nurse performance in the presence of moderators and mediators at the same time. This study empirically enlightened that the mediation impact of self-efficacy among paternalistic leadership and performance is strengthened in high-power distances.

## Data Availability Statement

The original contributions presented in the study are included in the article/supplementary material, further inquiries can be directed to the corresponding author/s.

## Ethics Statement

The studies involving human participants were reviewed and approved by Ethics Committee, CUST. Written informed consent for participation was not required for this study in accordance with the national legislation and the institutional requirements.

## Author Contributions

All authors listed have made a substantial, direct, and intellectual contribution to the work, and approved it for publication.

## Conflict of Interest

The authors declare that the research was conducted in the absence of any commercial or financial relationships that could be construed as a potential conflict of interest.

## Publisher’s Note

All claims expressed in this article are solely those of the authors and do not necessarily represent those of their affiliated organizations, or those of the publisher, the editors and the reviewers. Any product that may be evaluated in this article, or claim that may be made by its manufacturer, is not guaranteed or endorsed by the publisher.
